# Study of Antioxidant Properties of Agents from the Perspective of Their Action Mechanisms

**DOI:** 10.3390/molecules25184251

**Published:** 2020-09-16

**Authors:** Alla Ivanova, Elena Gerasimova, Elena Gazizullina

**Affiliations:** Chemical Technological Institute, Ural Federal University Named after the First President of Russia B. N. Yeltsin, 620002 Ekaterinburg, Russia; e.l.gerasimova@urfu.ru (E.G.); e.r.gazizullina@urfu.ru (E.G.)

**Keywords:** antioxidant capacity, electron-transfer-based assays, hydrogen atom transfer-based assays, chelating-based assays, electrochemical methods

## Abstract

The creation and analysis of a large variety of existing methods for the evaluation of integrated antioxidant properties are quite relevant in connection with a range of biological mechanisms of the antioxidants (AO) action. In this work, the existing methods are correlated with mechanisms of antioxidant action. It is shown that the results obtained by various methods are mainly incomparable. This can be connected with the implementation of various mechanisms of antioxidant action in methods. The analysis of the literature data presented in this review indicates the difficulty of creating a universal method and the feasibility of using integrated approaches based on the use of several methods that implement and combine various mechanisms of the chemical conversion of antioxidants. This review describes methods for studying the chelating ability of antioxidants, except for methods based on electron and hydrogen atom transfer reactions, which are currently not widely covered in modern literature. With the description of each mechanism, special attention is paid to electrochemical methods, as the interaction of active oxygen metabolites of radical and non-radical nature with antioxidants has an electron/proton/donor-acceptor nature, which corresponds to the nature of electrochemical methods and suggests that they can be used to study the interaction.

## 1. Introduction

Although the topic of antioxidants (AO) research dates to the beginning of the last century, it is still relevant [1,2,3]. This is due to the great variety of biological mechanisms of antioxidant action in the human body [4,5,6,7,8] and, accordingly, the lack of possibility to create a universal method of antioxidant research. One of the relevant directions in this area is the creation of approaches to the determination of integral parameters of antioxidant and antiradical capacity/activity because the efficiency of the body’s antioxidant system may not be related to the content of a particular compound, but reflects the property of the system as a whole. Many researchers have attempted to systematize the existing integral methods of studying antioxidants [9,10,11,12,13,14,15], classifying them by the method used, the nature of the analytical signal formation, trying to get as close to the conditions in vivo as possible, etc. Thus, this topic has not subsided until now, as shown by the appearance of new review works [16,17,18,19].

From our point of view, the methods should be classified according to the AO transformation nature in selected model systems instead of the analytical signal formation nature and/or the registration method. In addition, the creation or search of a unified universal approach does not seem to be a promising direction of development in the antioxidant research field. One of the most important things that has been done in recent works is an attempt to correlate existing methods with mechanisms of antioxidant action in the human body [19,20,21,22,23]. In this case, many of them [24,25,26,27,28,29,30,31,32,33] can be combined into a fairly coherent systematic picture in which each method will take its place and will be able to predict one of the mechanisms of antioxidant action in the human body. This is what we want to do as part of this work.

Taking the above into account, in this review, the methods will be classified in terms of mechanisms of antioxidant action in biological medium.

## 2. Basic Principles of Classification of Analytical Methods

Mechanisms of formation and biological action of reactive oxygen species (ROS) in living systems are the main element of a number of physiological and pathophysiological processes [34,35,36]. In fact, the organism’s protection from ROS is a universal complex system of chemical and biochemical reactions realized at different biological levels and proceed in accordance with different mechanisms, which involve various high and low molecular weight compounds, such as redox enzymes, polypeptides, and some vitamins, amino acids, polyphenols, etc.

**Figure 1:**
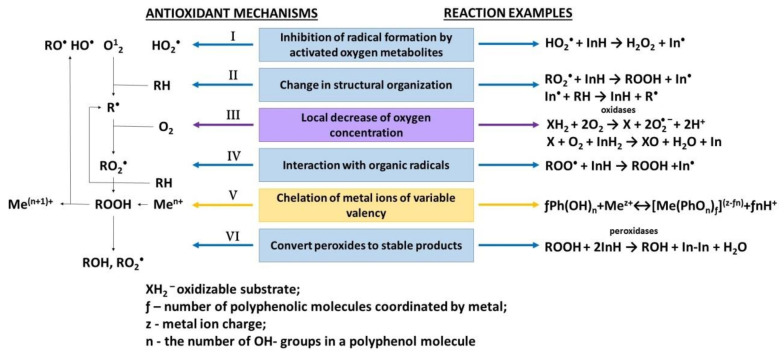
Mechanisms of biological action of antioxidants.

As a rule, six basic mechanisms are described in the literature, which are shown in Figure 1, considering the action of AO in biological media [4,5,6,19,20,21,22,37,38].

These mechanisms of biological action, in turn, from the chemical point of view of AO conversion are reduced to three main mechanisms:

(i) electron transfer reactions from AO to the substrate (AO oxidation reaction), i.e., ET-mechanism;
(ii) transfer reactions of a hydrogen atom from AO to a substrate which, in aqueous media, can be considered as proton transfer accompanied by electron transfer (AO oxidation reaction), i.e., HAT-mechanism;
(iii) transfer reactions of one or more electron pairs with the formation of the covalent bond by the donor-acceptor mechanism (the reaction of the complexation of AO with metal ions of variable valency), i.e., chelating-mechanism.

As a rule, the ET-mechanism does not occur in the body in its pure form because the proton environment is present in the living organism. Therefore, the mechanism of biological action III (Figure 1) is more correct to be considered as a combination of ET/HAT-mixed mechanism.

**Figure 2:**
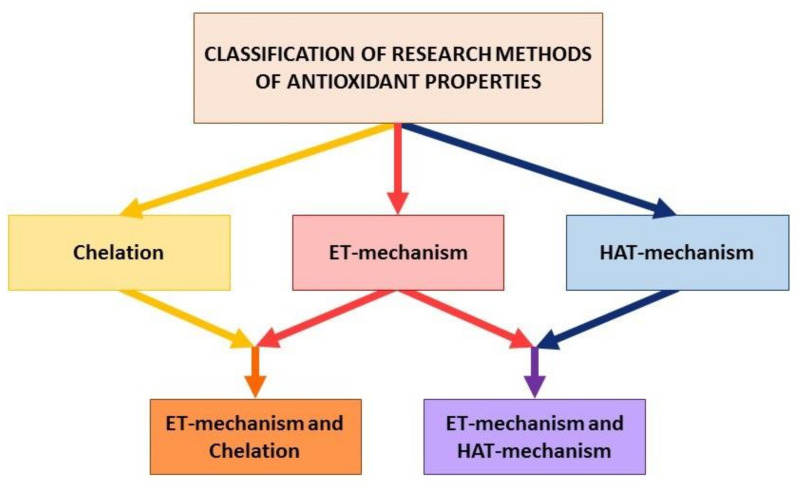
Classification of methods to evaluate the integrated antioxidant properties.

Thus, existing methods can be classified according to the scheme shown in Figure 2 based on the three main types of reactions.

The same AO can undergo chemical transformation either by one of three mechanisms, or by two mechanisms simultaneously depending on the compound structure, the environment, and various other conditions. The mechanisms variety of biological action and chemical transformation entails a variety of developed methods to study the antioxidant properties of compounds. We will review various methods for the integral assessment of antioxidant properties known from the literature in accordance with these mechanisms.

## 3. Methods to Evaluate Integrated Antioxidant Properties

### 3.1. Electron-Transfer-Based Assays

Antioxidants studies based on electron transfer reactions, the so-called ET (Electron-transfer), demonstrate the ability of an antioxidant to transfer an electron to reduce metal ions, carbonyls, and radicals [19,20,21,22]. The ET-mechanism of antioxidant action can be summarized in the following reactions (1)–(3):

ROO^•^ + AH/ArOH → ROO^−^ + AH^•+^/ArOH (1)

AH^•+^/ArOH^•+^ + H_2_O ↔ A^•^/ArO^•^ + H_3_O^+^ (2)

ROO^−^ + H_3_O^+^ ↔ ROOH + H_2_O (3)

**Figure 3:**
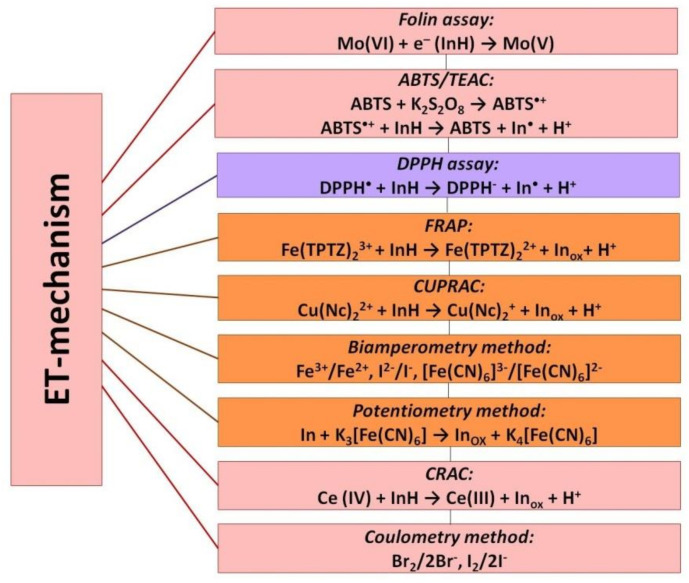
Electron-transfer-based assays.

As previously noted, ET-reactions in their pure form are rare, and proton transfer reactions usually follow electron transfer reactions. Nevertheless, the variants of integral assessment of antioxidant properties based on the electron transfer reaction represent the most numerous group in which the antioxidant effect of the studied samples (model substances or real samples) is modeled using an oxidizing agent. Most likely, this is due to the fact that Electron-transfer-based assays can be more easily implemented in analytical practice. In assays based on electron transfer reactions thermodynamic conversion (oxidation) of AO is measured at the end of the redox reaction or over a certain period. In fact, they are aimed at measuring the reducing ability of compounds, which, although not directly related to the ability of AO to inhibit ROS, is nevertheless a very important parameter because it reflects its redox characteristics and the thermodynamic possibility of interaction with ROS. Main widespread methods are presented in Figure 3.

#### 3.1.1. Spectroscopic Methods

Spectroscopic methods are widespread due to a number of undeniable advantages [19,20,21,39]. In most of the methods widely represented in the literature, AOs reacts with a fluorescent oxidizing agent or a colored oxidizing agent, which leads to a decrease in the intensity of reagent absorption. The decrease degree of an optical density at a given wavelength correlates with the concentration of AO in the sample.

##### Folin-Ciocalteu Assay

The well-known Folin-Ciocalteu reagent is used to determine the total content of phenolic compounds [40,41]. In the method, the increase of the optical density is measured after the interaction of the Folin-Ciocalteu reagent with various phenols. The reagent is hexavalent phosphomolybdic/phosphotungstic acid complexes (3H_2_O-P_2_O_5_-13WO_3_-5MoO_3_-10H_2_O). Sequences of reversible one- or two-electron reduction reactions lead to blue coloring of the solution. The general form of the reaction can be expressed as follows (4):

Mo(VI) (yellow) + e^−^ → Mo(V) (blue) (4)

Obviously, the Folin–Ciocalteu reagent is nonspecific for phenolic compounds and can be reduced by many non-phenolic compounds (for example, vitamin C, Cu (I), etc.). Phenolic compounds react with the reagent only under alkaline conditions (pH~10). The dissociation of the phenolic proton leads to the formation of a phenolate anion, which is also capable of reducing molybdenum. This fact once again proves the electron transfer mechanism. Colored compounds, formed between phenols and the reagent, are independent of the structure of phenolic compounds. The assay using the Folin-Ciocalteu reagent is convenient, simple, and reproducible with respect to the determination of the total phenol content despite the uncertain chemical nature. The serious limitations of this assay include the fact that obtained data cannot be unambiguously interpreted in relation to the body as the conditions of analysis are quite far from physiological.

##### ABTS/TEAC Assay

TEAC (Trolox equivalent antioxidant capacity). This assay is based on the reaction of the formation of ABTS^•+^ cation radicals from 2,2′-azinobis(3-ethylbenzothiazoline-6-sulfonate), which intensively absorbs at a wavelength of 734 nm. AOs of the investigating sample inhibit the color development of the resulting ABTS^•+^ (5):

ABTS^•+^ + InH → ABTS + In^•^ + H^+^ (5)

In general, the TEAC assay has many advantages which contribute to its wide application in screening the antioxidant capacity of a wide range of agents. The TEAC assay involves express, simple reactions with ABTS^•+^ proceeding quickly and in a wide pH range. ABTS^•+^, being a single charged positive radical, is soluble in both aqueous and organic solvents and is not affected by the ionic strength of a solution. Therefore, it can be used in various media to study the AOs properties of lipophilic and hydrophilic compounds. The reactions on which the assay is based can be automated or adapted to microplates [42] as well as to flow-injection analysis [43].

However, the TEAC assay also has some serious disadvantages. The high molar absorption coefficient of the ABTS^•+^ (1.50 × 10^4^ L mol^−1^ cm^−1^ at 734 nm [44]) limits the range of studied concentrations of antioxidants which can be quite accurately determined by the optical method (low detection limit—1.570 μM, narrow range of linearity of optical density). In addition, in order to obtain reproducible results, many reaction factors must be carefully monitored: the oxidizing agent used to generate ABTS^•+^; time and storage conditions of ABTS^•+^ solution; temperature of analysis; oxygen concentration in solution. All of these factors can affect the reliability of results.

##### DPPH Assay

DPPH (1,1-diphenyl-2-picrylhydrazyl) is a stable radical with a deep violet color, the reaction of which with other radicals or reducing agents leads to a decrease of absorption at 515 nm [45,46] (6).

DPPH^•^ + InH → DPPH^−^ + In^•^ + H^+^ (6)

The assay is the simplest and inexpensive one due to which it has become popular and widely used in various laboratories. The DPPH assay is technically simple, but some disadvantages severely limit its use. The DPPH is a long-lived radical that does not resemble the highly reactive peroxyl and other oxygen radicals involved in oxidative reactions in the body. Many AOs that quickly react with peroxyl radicals may react slowly with DPPH or may even be inert to it. It should be noted that DPPH^•^ is a stable radical and, just as ABTS^•+^ radical, is not an exact model of radical reactions in biological systems. It should not go unmentioned the effect of pH. The pH change leads to a significant change in the ionization equilibrium of phenols and affects the value of the reaction rate constant [47].

##### FRAP Assay

In the FRAP assay (Ferric reducing antioxidant power assay), complex salts of Fe(III) are used as an oxidizing agent, most commonly [Fe(TPTZ)_2_]Cl_3_, (TPTZ ligand 2,4,6-tripyridyl-triazine), also with phenanthroline and ferricyanide ions [48]. So, [Fe(TPTZ)_2_]Cl_3_ is reduced to [Fe(TPTZ)_2_]Cl_2_ during electron transfer from AO. [Fe(TPTZ)_2_]Cl_2_ intensively absorbs at a wavelength of 593 nm.

Fe(TPTZ)_2_^3+^ + InH → Fe(TPTZ)_2_^2+^ + In_ox_ + H^+^ (7)

FRAP assay is quite simple to implement. However, the presence of free ions of variable valence metals, in particular Fe (III), can lead to potential problems since real samples (biological samples and food products) contain a large number of compounds capable of chelating metal ions. It can affect the measurement error of the optical density. In addition, FRAP assay is implemented in the acidic environment at pH 3.6 which imposes at least the following limitations on the assay: a strong deviation from physiological conditions of a body and a decrease of the reactivity of phenolic AO. Also, FRAP assay does not allow the determination of thiol antioxidants (cysteine and glutathione) [49,50], which are important components of the antioxidant system of the body defense.

##### CUPRAC Assay

The assay of determining the antioxidant capacity [51] is based on measuring the absorption of the chromophore complex Cu (I) with neocuproin (Nc, 2, 9-dimethyl-1, 10-phenanthroline) formed as a result of the redox reaction of AO with the reagent CUPRAC (Cu complex (II) with neocuproine [Cu(Nc)_2_]^2+^. The absorption maximum of the formed complex [Cu(Nc)_2_]^+^ is recorded at a wavelength of 450 nm.

[Cu(Nc)_2_]^2+^ + InH → [Cu(Nc)_2_]^+^ + In_ox_ + H^+^ (8)

where InH is an antioxidant; In_ox_ is an oxidation product of the antioxidant.

CUPRAC assay is successfully used to measure the antioxidant capacity of food products and biological samples. CUPRAC assay features can be summarized as follows [52,53]:

- Reagent CUPRAC quickly enough reacts with a wide range of AOs including thiol compounds;
- CUPRAC reagent is sufficiently stable and can be immobilized on the surface of the cation exchange polymer to create an inexpensive sensor;
- The redox reaction is carried out at a pH close to physiological (pH 7 ammonium acetate buffer) in contrast to conditions of other assays (pH 3.6 for the FRAP or pH 10 in the Folin assay). The reducing ability of AO decreases under more acidic conditions than physiological pH due to the AO protonation. In alkaline media, the AO oxidation mechanisms differ from their in vivo transformations.

CUPRAC assay, as other optical methods, has a number of limitations associated with the possible imposition of absorption spectra of the oxidizing agent and the studied samples, i.e., there are difficulties with the analysis of colored samples in the visible region, and there are difficulties with the analysis of colourless agents in the UV region.

##### CRAC Assay

CRAC assay (*Ceric Reducing Antioxidant Capacity*) is based on the AOs oxidation study by cerium (IV) sulfate in diluted sulfuric acid at room temperature (9).

Ce^4+^ + InH → Ce^3+^ + In_ox_ + H^+^ (9)

Spectrophotometric determination of the remaining Ce (IV) is carried out after completion of the antioxidant reaction at a wavelength of 320 nm [54]. Quercetin and gallic acid, respectively, are used as standards for the study of flavonoids and phenolic acids, and the results of antioxidant measurements are calculated in trolox equivalents. The disadvantages of this assay include all limitations inherent to optical methods [55]. In addition, Ce^4+^ is a strong oxidizing agent. The potential is from 1.40 to 1.73 V (depending on the environment) which exceeds the redox potential values of some ROS.

#### 3.1.2. Electrochemical Methods

It can be seen from the above-mentioned examples that in the process ET-methods descripting and developing a great attention is paid to optical methods based on oxidation reactions of AOs with electron transfer, while the mechanism of this process has an electrochemical nature. In fact, the signal-forming reaction in ET methods is a rather simple redox reaction between an oxidizing agent and an AO which in general case can be represented as follows (10):

Oxidizing agent _(ox)_ + e (from AO) = Oxidizing agent _(red)_ + AO_(ox)_ (10)

Therefore, it is logical that electrochemical methods should be used to study the process of electron transfer.

Electrochemical methods are characterized by simplicity of use, low cost of equipment, rapidity, low detection limit [56,57,58,59,60], and the possibility for the implementation of both portable and flow analysis variants [61,62,63].

Traditionally, direct electron transfer from an AO to an electrode can be used and the cyclic voltametry (CV), the differential pulse voltametry (DPV) and the squarewave voltametry (SWV) used to study the electrochemical behavior of phenolic compounds are covered in various reviews [64,65,66,67]. However, problems associated with occurrence of heterogeneous electrochemical reactions, especially in the study of complex and multicomponent samples, often lead to distortion of voltammograms and complicate the results interpretation. The purpose of this review is to consider methods based on the use of an oxidizing agent in electron transfer reactions.

##### Coulometry

Coulometric methods of studying AO with electrogenerated titrants were successfully developed. They are also based on the reaction of electron transfer from AOs to oxidizing agents obtained as a result of electrolysis under certain conditions [68].

Halogens are mainly used as model oxidizing agents [69,70,71,72,73,74,75], but in recent years there have been published articles concerning electric generation of other model oxidizing agents, in particular iron hexacyanoferrate [76]. The coulometry method with electro-generated titrants has a great advantage over other methods because it is standardless, does not require the preparation of standard solutions, and therefore does not require storage. It is possible to achieve very high measurement accuracy provided that 100% current efficiency is observed when generating an oxidizing agent.

However, not only oxidation-reduction reactions with AOs are possible, but also reactions of electrophilic addition to multiple bonds and substitution in the aromatic ring using electrogenerated halogens. This can lead to distortion of results.

##### Biamperometry

The biamperometric determination of integral parameters of antioxidant capacity includes the reaction of a test analyte with a redox pair such as Fe^3+^/Fe^2+^, I_2_/I^−^, [Fe(CN)_6_]^3−^/K_4_[Fe(CN)_6_]^4−^, DPPH^•^/DPPH [77,78,79]. A wide range of samples was studied: biological samples (urine, blood), food, and drinks using the proposed assay. For example, when using the DPPH^•^/DPPH pair, the measured current strength is limited by a lower radical concentration at the DPPH^•^ concentration lower than the DPPH concentration [79,80]. Adding an AO increases the DPPH concentration due to the reduction of the DPPH^•^ radical, thereby inducing a current linearly dependent on the AO concentration.

The variants of method implementation are described for the DPPH^•^/DPPH system using both two identical Pt electrodes and glassy carbon electrodes. In addition, biamperometric assays using the ABTS^•+^/ABTS system are known [81,82].

However, biamperometry requires strict control over experimental conditions, in particular, the potential applied must be thoroughly controlled.

##### Potentiometry

Analytical signal in this method is the potential shift of the platinum electrode in the K_3_[Fe(CN)_6_]/K_4_[Fe(CN)_6_] system which is observed upon the introduction of the analyzed sample [83,84,85,86,87].

This shift is the ration change consequence of oxidized and reduced forms as the reaction result (11):

n[Fe(CN)_6_]^3−^ + In = n[Fe(CN)_6_]^4−^ + In_Ox_ (11)

The method has been tested for a wide range of agents and has a number of features:

1. the potassium hexacyanoferrate (III) is an oxidizing agent of medium strength (E° = 0.36 V), i.e., there is a thermodynamic possibility of the reaction of the oxidizing agent with most antioxidants having, by definition, low oxidation potentials. In this case, there is no possibility of interaction of the potassium hexacyanoferrate (III) with compounds having weak reducing properties and not related to antioxidants;
2. the reaction of the potassium hexacyanoferrate (III) with AOs can be realized under conditions close to physiological, i.e., at pH close to 7 (values of the conditional stability constants of potassium hexacyanoferrate (III) and its reduced form, potassium hexacyanoferrate (II), at pH = 7 significantly exceed 10^8^);
3. potassium hexacyanoferrate (III) is a one-electron electron acceptor, its reaction with AOs proceeds stoichiometrically in accordance with the number of functional groups exhibiting antioxidant properties, and the results are expressed in universal units of measurement—mol-eq/l (M-eq);
4. potentiometric measurements suggest the possibility of investigating any samples including turbid and colored ones.

Thus, not all existing analysis methods can be clearly classified as ET-mechanism based. In fact, most of these methods are implemented on a mixed mechanism:

- the TEAC and the DPPH assays are based on the transformation of a stable radical into an inactivated form according by the antioxidant mechanisms of ET or HAT;
- methods based on the use of metals and their complexes as oxidizing agents the (FRAP, CUPRAC, CRAC, the Potentiometry assay with K_3_[Fe(CN)_6_]) are implemented by the mixed mechanism (ET + Chelating), since many antioxidants are capable of forming sufficiently strong complexes with metals of variable valency [37,38].

### 3.2. Hydrogen Atom Transfer-Based Assays

The antioxidant’s ability to “quench/intercept” free radicals by detaching a hydrogen atom is measured in assays based on hydrogen atom transfer reactions. The mechanism of this type of reactions is reduced to the transfer of a hydrogen atom from an antioxidant to the radical ROO^•^ (12):

ROO^•^ + AH/ArOH → ROOH + A^•^/ArO (12)

**Figure 4:**
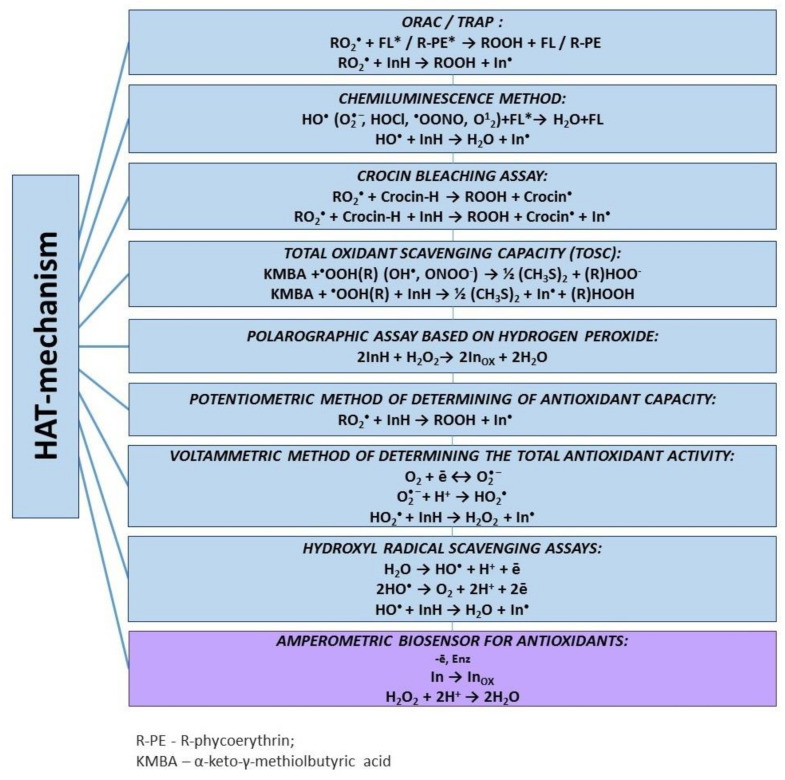
Hydrogen atom transfer-based assays.

Effective antioxidants must react with free radicals faster than biomolecules, thus protecting the latter from oxidation. The main methods based on the HAT-mechanism are presented in Figure 4.

In this group of methods, radical-generating systems are most often used as oxidizer agents models (2,2′-azobis(2-amidinopropane)dihydrochloride (AAPH), azobisisobutyronitrile (AIBN), 2,2′-azobis[2-(2-imidazolin-2-yl)propane] dihydrochloride (AIPN) azoinitiators, peroxyl radicals are formed as a result of their thermal decomposition). They are considered to be biologically closer to the reactive oxygen radicals formed in the body [88,89]. In addition, there are assays based on studying the damaging effect of such ROS as O_2_^•−^, H_2_O_2_, HO^•^, ^1^O_2_, and ONOO^−^ which are more specific. Therefore, some authors distinguish them into a separate group of methods while according to the mechanism of interaction with AO, they can be attributed to the methods based on the hydrogen atom transfer reaction [90].

#### 3.2.1. Spectroscopic Methods

Spectroscopic methods have historically prevailed in the HAT-mechanism, as well as in electron transfer-based assays. The most common methods include ORAC, TRAP, and the Crocin bleaching assay. In most methods widely represented in the literature, AOs react with a fluorescent or colored oxidizing agent which leads to a decrease in the absorption rate of the reagent. The decrease degree of the optical density at a given wavelength correlates with AO concentration in the sample.

##### ORAC Assay

In the ORAC assay (oxygen radical absorption capacity), the AAPH azoinitiator is typically used as a model of generating radicals. Peroxyl radicals are formed as a result of thermal decomposition of AAPH by the reaction (13):

R-N=N-R+2O_2_ → 2RO_2_^•^+N_2_ (13)

the ROO^•^ subsequently react with a fluorescent agent (β-phycoerythrin or fluorescein) (14).

ROO^•^ + FL* → ROOH + FL (14)

If the sample contains AO, the fluorescence decreases due to the consumption of peroxyl radicals, and the antioxidant capacity is determined by comparing the fluorescence decrease in the presence and absence of antioxidants. Curves of fluorescence intensity vs. time are recorded, and the area under the curves (AUC) with and without addition of an antioxidant is calculated and compared to a standard curve generated using the standard antioxidant, trolox equivalent [91].

Modifications of the ORAC assay are distinguished depending on the model reaction of radical generation, for example, on the generation model of hydroxyl radicals [92]. The method has been successfully used for a wide range of samples: blood plasma [93], biological materials, and food products [94,95,96]. However, the disadvantages of ORAC assay include the lack of universal units of measurement, the lack of the interaction kinetics description of radicals with fluorescein, and radicals with antioxidants under experimental conditions.

##### TRAP Assay

The TRAP assay (total radical trapping parameter) determines the ability of antioxidants to inhibit the reaction between peroxyl radicals and a fluorescent agent. Assays related to TRAP analysis can be considered as variants of the ORAC assay in which the range of radical initiators and fluorescent agents are expanded. The thermal decomposition reaction of AAPH (a source of peroxyl radicals) [97,98,99], enzymes such as horseradish peroxidase [100], the H_2_O_2_-hemin system (a source of hydroxyl radicals) [101], NO^•^ [102], or singlet oxygen [103] can also be used as a source of free radicals/ROS. Dichlorofluorescein diacetate [104], fluorescein [103], phycoerythrin (R-phycoerythrin) [105,106], luminol [100], and ABTS [107] are used as fluorescent agents in TRAP assays.

The antioxidant capacity of samples is measured by comparing the induction periods obtained in the presence and absence of Trolox, the standard antioxidant. The induction period is calculated by extrapolating curves of the maximum oxidation of R-PE before and after the introduction of Trolox. It has been established that the use of dichlorofluorescein as a fluorescent agent is accompanied by side hydrolysis processes [108]. It should also be noted that TRAP assays have the same limitations as ORAC methods.

##### Chemiluminescence Assay

There is a large number of various modifications of the chemiluminescent method [109,110,111,112,113,114]. The most common modification of the chemiluminescent method for determining total antioxidant capacity is based on the use of luminol (LH_2_) as an activator of chemiluminescence. A sample is added to the reaction mixture with the addition of luminol, hydrogen peroxide, and a compound capable of forming radicals as a result of spontaneous thermolysis, for example, the AAPH.

Further, the peroxyl radical oxidizes luminol (LH_2_) to form the luminol radical (15):

ROO^•^ + LH_2_ → ROOH + LH (15)

Chemiluminescence is observed as a result of the molecule formation of the final product of the luminol oxidation, aminophthalic acid in the electronically excited state, which emits a photon, from LH^•^ through the formation of intermediate substances (16):

LH^•^ → P* → P + *hv* (16)

The chemiluminescence intensity is proportional to the photon production rate, and in turn, it is proportional to the stationary concentration of LH^•^ in the system. Interacting with radicals, antioxidants interrupt the described chain and prevent photon formation (17):

ROO^•^ + InH → ROOH + In^•^ (17)

Possible sources of radicals in the analysis of the antioxidant capacity of the sample using the chemiluminescent method are horseradish peroxidase—hydrogen peroxide, hemin—hydrogen peroxide, cytochrome c—cardiolipin—hydrogen peroxide [115,116,117], etc.

It should be noted that the chemiluminescent method is quite complex, time-consuming, and multi-stage. However, this method is one of the few approaches that allow not only to study antiradical properties of compounds of different nature, but also to evaluate the kinetic characteristics of free radical generation and inhibition which is a complete and comprehensive approach to the study of radical oxidation processes.

##### Crocin Bleaching Assay

The “competition kinetics” method [118] is known which uses the crocin oxidation by peroxyl radicals resulting from the decomposition of AAPH (1819–).

ROO^•^ + Crocin-H → ROOH + Crocin^•^ (18)

ROO^•^ + Crocin-H + InH → ROOH + Crocin^•^ + In^•^ (19)

In this case, the oxidation rate of crocin by peroxyl radicals is recorded in the absence and the presence of antioxidants by a change in the absorption intensity (λ = 450 nm). This method is one of the variations of the TRAP assay which also uses Trolox as its standard.

The β-carotene bleaching assay is referred to similar methods. This method is suitable for evaluating the antioxidant activity of essential oils and other lipophilic compounds. The method is based on a decrease in the optical density of β-carotene (λ_max_ = 470 nm) as a result of its reaction with the radicals formed by the oxidation of linolenic acid in the emulsion. The carotene oxidation is suspended when antioxidants are added. However, the bleaching of β-carotene at 470 nm may occur in several ways. Therefore, in some cases, there may be ambiguous interpretation of the data.

#### 3.2.2. Electrochemical Methods

Despite the obvious advantages of electrochemical methods related to the similar nature of the reactions, availability, simplicity, informativeness and low cost, the key reviews on hydrogen atom transfer-based assays pay little attention to them. At the same time, they are a worthy alternative to optical methods and their application can be quite diverse. Electrochemical methods can be used not only as methods for obtaining an analytical signal, but also as an assay to generate models of a radical nature.

##### Voltammetric Assay

There is a voltammetric method for determination of the total antioxidant activity where the process of oxygen electroreduction is used as a model reaction which is realized in several stages with the generation of reactive oxygen species on the electrode surface [119] (2021–):

O_2_ + *e ↔* O_2_^•^^−^ (20)

O_2_^•^^−^ + H^+^ = HO_2_^•^ (21)

**Figure 5:**
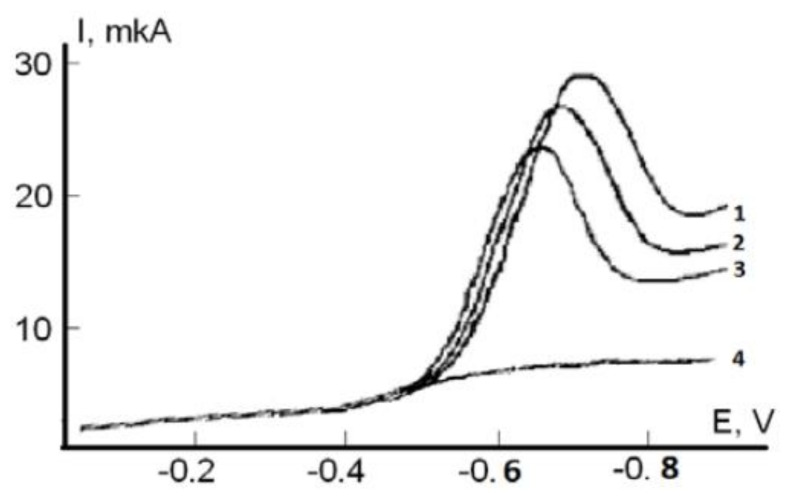
Voltammograms of the ER O_2_ current in phosphate buffer (0.025 M, pH 7.3) without (1) and with 0.2 mL of serum blood at t = 5 min (2), t = 10 min (3). (4) is the residual current without oxygen in the solution.

The decrease of the molecular oxygen reduction current and the shift of the process potential to the region of positive values are the reaction result of AO with the formation of oxygen radicals (Figure 5) cited from [120,121].

The interaction of AO with protonated superoxide anion radical occurs in the transfer mechanism of the hydrogen atom (22):

HO_2_^•^ + InH → H_2_O_2_ + In^•^ (22)

Capacitive and kinetic criteria were introduced to evaluate the antioxidant properties by the voltammetric method [122]. The capacitive criterion shows the process intensity decrease of oxygen electroreduction when AO is introduced into the background electrolyte solution. The kinetic criterion shows how many oxygen radicals are inhibited by samples AOs per unit time.

The developed method has been used to study a wide range of individual AOs of various chemical nature (polyphenols, vitamins), as well as extracts from plant materials and biological samples [123,124].

Disadvantages of the proposed method include the fact that the lifetime of the superoxide anion radical in the near-electrode space in aqueous media is quite short, and therefore it is preferable to use aprotic media for research [125].

##### Potentiometric Assay

**Figure 6:**
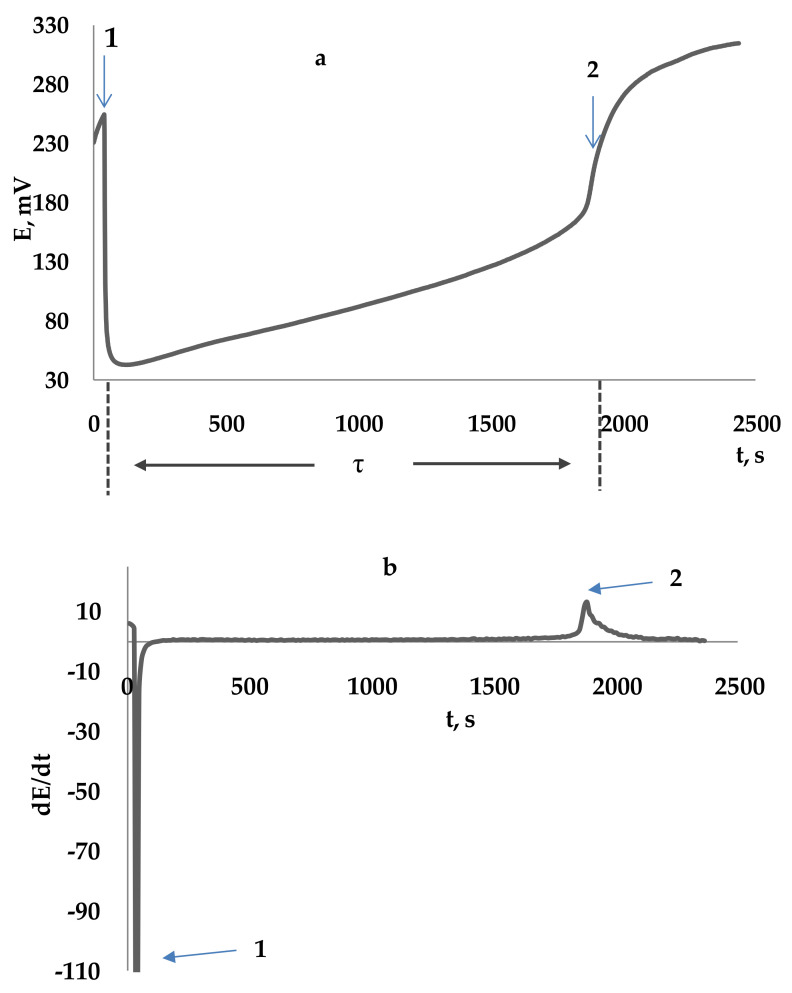
The kinetic curve of the potential change (**a**) and the derivative of the potential dependence on time (**b**): 1—0.1 M AAPH; 2—the addition of ascorbic acid (C = 0.3 mM) to 0.1 M AAPH.

The basis for the definition of total antiradical capacity (TAC) is a regular change of the redox potential [126,127]. Figure 6a,b shows kinetic curve of the potential change (a) and the derivative of the potential dependence on time (b): 1, 0.1 M AAPH; 2, the addition of ascorbic acid (C = 0.1 mM) to 0.1 M AAPH. The regular change of potential is due to the following processes:

- (1) initiating a radical reaction by thermostating the initiator’s solution AAPH. A potential growth is observed in the system (the portion of the curve up to point t_1_ due to the generation of an oxidizing agent (peroxyl radicals));
- (2) the addition of oxidation processes inhibitors InH (t_1_) reacting with peroxyl radicals, increases the chain break in reactions (23)–(25):

RO_2_^•^+ InH → ROOH + In^•^ (23)

RO_2_^•^+ In^•^ → molecule product (24)

In^•^+ In^•^ → product (25)

The introduction of a reducing agent into the system is accompanied by decrease in its potential. Completion of the induction period, indicating the depletion of antioxidants in the system, is accompanied by a sharp increase in potential (designation t_2_ in Figure 6a). The induction period determined as the time from the introduction of the antioxidant into the initiator solution to the point corresponding to the maximum rate of change in the potential (dE/dt)_max_, which is defined as the maximum of the derivative function in the dependence of the redox potential on time (Figure 6b).

Determination of the coefficient (f) showing how many chain breaks occur per one inhibitor molecule according to the Formula (26):

*f* = (τ·W_i_)/C_InH_ (26)

Determination of the total antiradical capacity (TAC) using the Formula (27):

TAC = *f*·C_InH_ = W_i_·τ (27)

where TAC is the total antiradical capacity, M-eq; τ is the induction period, s.

The proposed assay can serve as an alternative to the widely used ORAC/TRAP assays, since it is simpler to implement, does not require the presence of additional fluorescent agents, and therefore additional reaction stages. This assay uses direct interaction of the antioxidant with peroxyl radicals, due to the absence of additional reaction processes, the data obtained can be interpreted more clearly. In addition, the assay has simple and available potentiometric detection.

##### Amperometric Biosensors

**Figure 7:**
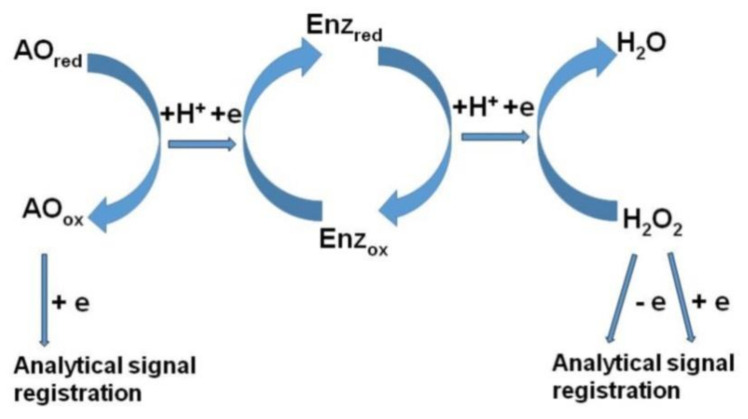
The scheme of amperometric response of peroxidase-based biosensors to compounds with antioxidant properties or to hydrogen peroxide (Enz—enzyme, AO—antioxidant).

There is a group of amperometric enzyme biosensors to determine the total antioxidant properties of the studied samples. Figure 7 presents a general view of the analysis scheme.

In these assays, the amperometric response can be detected both by the reduction current of the oxidized AO and by the kinetics of hydrogen peroxide consumption. Hydrogen peroxide refers to ROS, and the reaction of hydrogen peroxide with AO also proceeds via the proton-electron transfer mechanism, but with the participation of redox enzymes.

Amperometric biosensors are successfully used to determine the total content of polyphenols in food products (tea, wine, etc.). The content of phenolic AO is expressed in units of catechin [128,129] or chlorogenic acid [130]. It is shown that the total content of phenolic AOs correlates with the total antioxidant capacity measured on the DPPH stable radical model [130].

Total antioxidant properties can also be assessed by the kinetics of hydrogen peroxide consumption using amperometric enzyme sensors. However, a common significant disadvantage of these sensors is the need for enzymes. On the one hand, enzymes are an integral part of the antioxidant defense system of the body, on the other hand, their instability and high cost limit the widespread use of such methods.

The amperometric method for determining AOA, where hydrogen peroxide is also used as an oxidizing agent, was proposed by the authors [131]. Prussian blue (iron hexacyanoferrate) is used instead of enzymes. The authors show advantages of the proposed biosensor over enzyme biosensors. Advantages include an increase of three orders of magnitude of the rate constant of oxidation and reduction of hydrogen peroxide and an increase of three orders of magnitude of selectivity with respect to the reduction of hydrogen peroxide [132]. Information about AOA is obtained by measuring a decrease of the reduction current of hydrogen peroxide. The obtained results agree well with the standard method on the model of lipid peroxidation, but the new proposed approach is easy to use and low cost which distinguishes it from enzymatic analogues.

##### Hydroxyl Radical Scavenging Assays

The authors [133,134] proposed a method to study the antioxidant properties on the model of electro-generated hydroxyl radicals (Hydroxyl radical (HO^•^) scavenging assays) via the electrooxidation of water according to Equations (28) and (29):

H_2_O → HO^•^ + H^+^ + e^−^ (28)

2HO^•^ → O_2_ + 2H^+^ + 2e^−^ (*k*_2_) (29)

In this case, hydroxyl radicals are formed as intermediate species adsorbed on the anode surface. The formation rate of hydroxyl radicals is determined by the current density of electrolysis. The adsorption degree of these radicals on the anode surface depends on the anode material.

Information regarding the scavenger activity of antioxidants is obtained by the relative value of the rate constant of the reaction between antioxidants and HO^•^ radicals, *k_AO;HO_*_•_/*k*_*O*2_. The number of HO^•^ radical scavenged per molecule of antioxidant can also be calculated.

The method has been successfully tested for known natural antioxidants and food products.

It should be noted that the electrochemical generation of radicals has several advantages over other methods. In addition to the well-known advantages of electrochemical methods, such as low cost and automation simplicity, the degree and speed of the generation reaction can be controlled by the nature of the anode material [135,136]. The method allows studying the kinetics of the interaction of hydroxyl radicals with AOs and their oxidation products, which is very important.

##### Polarographic Assay of Hydrogen Peroxide Scavenging

A polarographic method has been proposed to determine total AOA based on the consumption model of hydrogen peroxide [137,138] (30).

2InH + H_2_O_2_ → 2In_Ox_ + 2H_2_O (30)

**Figure 8:**
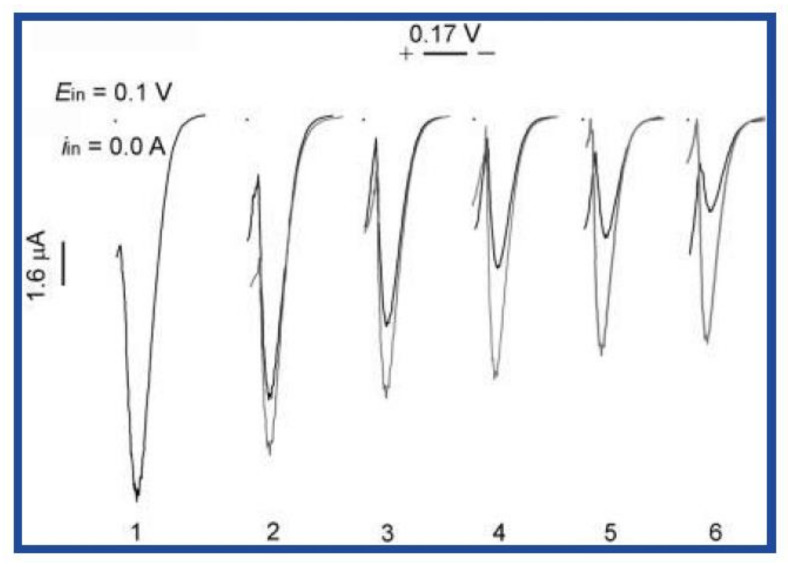
Anodic polarographic curves of H_2_O_2_ before (1) and after addition of red (**dark line**) and white wine (**gray line**): (2) 100, (3) 200, (4) 300, (5) 400 and (6) 500 μL.

Direct current polarography has been used to survey hydrogen peroxide scavenge (HPS) upon gradual addition of tested samples. Results expressed as reciprocal value of AO volume required for 50% decrease of anodic limiting current of hydrogen peroxide (Figure 8 cited from [138]).

The assay has been successfully applied for the analysis of a wide range of drinks. High degrees of correlation with the DPPH assay and Folin assay have been obtained [138,139].

### 3.3. Chelating-Based Assays

It has been shown earlier that a great attention of researchers is drawn to the creation of methods to assess the antioxidant properties of agents based on the implementation of two main mechanisms of the chemical action of AOs related to the electron transfer and hydrogen atom transfer. There is a third action mechanism associated with the ability of AO to form complexes with metal ions of variable valency. Effective chelators of metal ions of variable valency can also provide protection against oxidative damage to biological macromolecules, since free metal ions are involved in the HO^•^ generation reaction (reactions of the Fenton type: Fe(II) with hydrogen peroxide) (31). HO^•^ radicals are the most highly reactive radicals and contribute to the development of oxidative stress [140,141].

Fe^2+^ + H_2_O_2_ → Fe^3+^ + HO^•^ + HO^−^ (31)

Fe(III) ions can also participate in the reaction of radical formation from peroxides, although the rate of these reactions is an order of magnitude lower than with Fe(II) ions [142].

Studies have shown that the electron transfer reaction between the metal of variable valency and hydrogen peroxide occurs not by the mechanism of electron transfer from the outer sphere, but by the mechanism of electron transfer from the inner sphere, at which an intermediate complex of H_2_O_2_ with metals of variable valency is formed preceding the electron transfer process [143]. Therefore, if the metal of variable valency is coordinatively saturated, it does not react with the H_2_O_2_ to form the hydroxyl radical. It is known that some AOs of phenolic nature, containing catechol and gall fragments in their structure, can form stable coordination saturated complexes with metals of variable valency, thereby inhibiting oxidation processes at the stage of chain branching [37,38].

According to the scheme, iron ions can coordinate up to three polyphenol molecules. The values of the total stability constants for some natural phenols are from 13 to 46, which indicates their high complex-forming ability. For comparison, it can be stated that values of the stability constant of ions of some metals (Cu, Zn, Co, Fe, Mn) with the “classical” complexing agent ethylenediaminetetraacetic acid is from 13 to 19.

Thus, the study of the chelating properties of biocompounds can also serve as a good approach in the study of antioxidant properties. This is confirmed by numerous studies of the relationship between increased levels of free iron ions in humans and animals with increased risk of various diseases, such as vascular diseases, cancer, and some neurological conditions [144,145,146,147,148].

A key role in the study of chelating properties belongs to the study of iron complexation. Iron is an important element for all living cells and plays a vital role as a component of hemeproteins and as a coenzyme for nonheme iron enzymes [149,150]. On the other hand, Fe^2+^ ions are the most powerful prooxidants among various types of metal ions [151,152], such as copper, nickel, zinc, molybdenum, cobalt, and others. To provide protection against the toxic effects of free iron ions and its availability, the metal is bound to various peptides and proteins, the most important of which is iron-containing ferritin [149,153].

**Figure 9:**
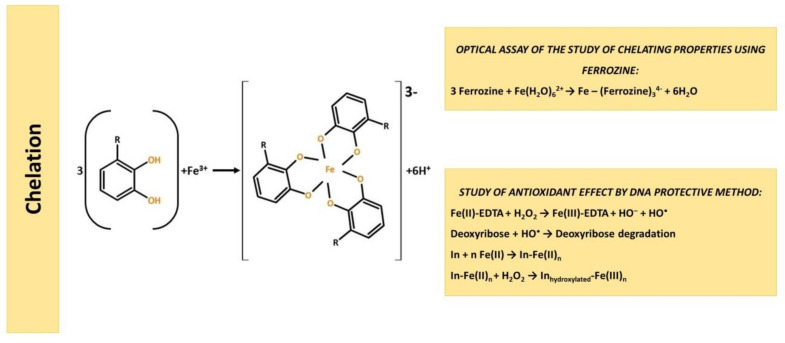
Chelating mechanism assays.

Main methods for determining antioxidant properties by the mechanism of iron chelating are shown in Figure 9.

#### 3.3.1. Chelating Properties Assay with Use Ferrozine

Measuring the Fe^2+^ ions concentration decrease can provide information on the chelating ability of substances.

The assay is based on the fact that in the presence of chelating agents, the formation of ferrozine complexes is disrupted, which leads to a decrease in the intensity of red color at a wavelength of 562 nm.

3 Ferrozine + Fe(H_2_O)_6_^2+^ → Fe − (Ferrozine)_3_^4−^ + 6H_2_O (32)

The absorption decrease measurement allows to evaluate the metal-chelating activity of a competing chelator. A lower absorption value indicates a higher metal chelating activity. Thus, the method is based on the implementation of competing complexation reactions in which antioxidant and ferrozine compete for iron ions.

Various compounds containing -OH, -SH, -COOH, -PO_3_H_2_, C=O, -NR_2_, -S-, and -O-functional groups, that are theoretically capable of exhibiting chelating ability, have been studied using this method [154,155,156]. A number of studies of the complexing properties of some natural polyphenols with high chelating ability has been described: cucurmin, quercetin, resveratrol, rosmarinic acid, etc. [157,158,159]. Complexation reactions differ in the mechanisms and stoichiometry of reactions depending on the structure of the AO.

Using a similar approach, the method for determining Cu^2+^ chelating ability using pyrocatechol violet (PV) as the complexing and chromogen agent is proposed [160].

#### 3.3.2. Antioxidant Assay by DNA Protective Method

Another assay to studying the chelating ability of AO is based on the study of the degree of DNA or deoxyribose damage by hydroxyl radicals generated by the Fenton reaction [161,162,163] (33)–(34).

Fe(II)-EDTA + H_2_O_2_ → Fe(III)-EDTA + HO^•^ + HO^−^ (33)

2-deoxyribose + HO^•^ → 2-deoxyribose degradation products (34)

The introduction of polyphenolic substances capable of chelating iron, such as tannic acid, forming stable complexes that cannot participate in Fenton reactions (35)–(36). Due to this, 2-deoxyribose degradation is significantly inhibited.

In + nFe(II) → Fe(II)_n_-In (35)

Fe(II)_n_-In + H_2_O_2_ → Fe(III)_n_-In + HO^−^ (36)

where In is an antioxidant.

The DNA damage degree change with the addition of polyphenolic substances is studied using the standard gel electrophoresis method. The deoxyribose damage degree is studied by spectroscopic determination of malondialdehyde (the product of oxidative degradation of deoxyribose under the action of hydroxyl radicals formed in the Fenton reaction). In this case, malonic dialdehyde is determined by the reaction with thiobarbituric acid.

The main advantage of an assay based on the determination of the deoxyribose damage product is a quick screening of compounds. However, conditions for implementing the method are far from biological system. In addition, many Fe-polyphenol complexes may absorb near the wavelength of the deoxyribose degradation product (at 532 nm), which makes this assay problematic for measuring the indirect effect of polyphenols on the degree of substrate degradation. From this point of view, gel electrophoresis, perhaps, has advantages, because this method directly determines DNA damage and the implementation conditions of the assay are close to biological (such as pH, buffer capacity, and ionic strength).

However, this approach is very indicative and allows to explain the antimutagenic and anticarcinogenic activity of many compounds by chelating of Fe(II) and the protection of DNA from oxidative damage.

Thus, this section shows the importance of studies of the chelating ability of AOs, which are based on the study of the transfer of electron pair from AOs to an oxidizing agent, where metal ions of variable valence indirectly act as an oxidizing agent. It should be noted that this assays group, in contrast to the other two groups described above, is given very little attention in the literature, while the chelating ability of AO is directly related to the protection of the body’s DNA from oxidative degradation induced by hydroxyl radicals formed in the Fenton reaction. It should also be noted that complexation reactions are very convenient to study by electrochemical methods, since these reactions are associated with changes in the electrochemical properties of the system, namely, the shift in redox (potentiometry [164,165,166,167]) or electrode potential (voltammetry [168,169,170]). Methods of potentiometry and voltammetry in the study of complexation reactions have been used for a long time and their application for research of chelating properties of AO is very promising, however such methods are not found in the literature. An attempt to describe the chelating ability of polyphenolic compounds has been made in the potentiometric method using potassium hexacyanoferrate (III). However, it has now been proven, and so far only qualitatively shown [171], that the obtained values of antioxidant capacity are due to the total effect of antioxidant action associated with both the electron transfer from AO to hexacyanoferrate and the chelating process.

## 4. Some Comparisons of Results Obtained Using Methods by Various Mechanisms

There is a great variety of methods based on both individual and mixed mechanisms, as described above. In addition, many methods use different units of results measurement, and this makes comparison difficult. The purpose of this chapter is to show that results obtained using methods based on various mechanisms can significantly differ in absolute values, while a different degree of correlation up to its absence can be observed between them. This situation is quite predictable and logical, as the methods are based on different mechanisms of antioxidant conversion and use different models of the oxidizing agent.

In particular, results differ significantly in the study of vegetable extracts by the Folin assay, the DPPH assay, the FRAP, the CUPRAC and the TEAC in absolute values [172,173,174,175]. This is predictable because the Folin assay, the FRAP, the CUPRAC are based on the ET-mechanism, and the DPPH assay and the TEAC are based on the ET/HAT-mixed mechanism. However, the correlation between the total content of polyphenols and antioxidant capacities of raw and studied vegetables was high (R^2^ = 0.9971 and 0.9705 for the FRAP and the DPPH assay, respectively). This may be due to the fact that main antioxidants in plant materials are polyphenols.

The study of biological samples is much more complicated. The blood serum was studied using various methods to compare results [176]. A sufficiently low correlation of results was found when comparing results obtained by the ORAC and the FRAP methods (R^2^ = 0.349). No correlation was found when comparing results obtained by the methods ORAC/TEAC and FRAP/TEAC.

It was shown that, when studying antioxidant properties of various samples by the potentiometry using potassium hexacyanoferrate (III) (ET/chelating-mechanism) and peroxyl radicals (HAT-mechanism) [171] as an oxidizing agent, in the case studies of individual compounds and aqueous extracts raw materials, results correlate with each other, although they differ in absolute values. In the study of complex conjugate structures, there is no correlation between methods.

This once again proves the necessity of a comprehensive and integrated approach using methods based on various mechanisms of antioxidant action to obtain complete and objective information about the antioxidant effect of the studied object.

## 5. Conclusions

The existing methods to evaluate the integrated antioxidant properties, classified by three main chemical mechanisms coupled with main pathways of the biological action of antioxidants in a body, have been considered. Despite a rather big variety of methods and assays based on the implementation of one of the chemical and a combination of various mechanisms, it is quite difficult to choose a universal method that can comprehensively evaluate antioxidant properties of agents. This is due to the variety of mechanisms of biological action of antioxidants in a body. Taking into account that the ability to transfer electrons, hydrogen atoms, and electron pairs from an antioxidant to a oxidizing agent or ROS does not always correlate with each other, an integrated approach is needed to obtain objective information about antioxidant properties of the studied object, which will be based on the use of several methods, implementing and combining various mechanisms of chemical transformation of antioxidants. In this regard, it should be noted that little attention is paid to methods of studying the chelating ability of antioxidants, based on the mechanism of electron pair transmission with the formation of strong complexes, while the chelating ability of AO is directly related to the protection of DNA from oxidative degradation caused by hydroxyl radicals formed in the Fenton reaction.

Thus, the creation of integrated approaches to the study of antioxidants is a promising direction. In addition, special attention should be paid to electrochemical methods of analysis, taking into account current trends in the development of medical and analytical chemistry, aimed at creating and developing personalized medicine and related portable devices. Electrochemical methods are rarely presented in classical reviews, while they most closely reflect the antioxidants action mechanism based on the transfer of an electron/electron pair. In addition, they are quite simple when taking measurements, affordable, and can be easily implemented in a miniature version.
